# Good Performance of Revised Scoring Systems in Predicting Clinical Outcomes of *Aeromonas* Bacteremia in the Emergency Department: A Retrospective Observational Study

**DOI:** 10.3390/diagnostics14020124

**Published:** 2024-01-05

**Authors:** Cheng-Yang Wen, Sung-Yuan Hu, Ming-Shun Hsieh, Shih-Che Huang, Chia-Hui Shen, Yi-Chun Tsai

**Affiliations:** 1 Department of Emergency Medicine, Taichung Veterans General Hospital, Taichung 407219, Taiwan; koduck10@gmail.com (C.-Y.W.); aa1239076@gmail.com (C.-H.S.); rosa87324@gmail.com (Y.-C.T.); 2 Department of Post-Baccalaureate Medicine, College of Medicine, National Chung Hsing University, Taichung 402, Taiwan; 3 Institute of Medicine, School of Medicine, Chung Shan Medical University, Taichung 40201, Taiwan; 4 School of Medicine, Chung Shan Medical University, Taichung 40201, Taiwan; cucu0214@gmail.com; 5 School of Medicine, National Yang Ming Chiao Tung University, Taipei 112304, Taiwan; edmingshun@gmail.com; 6 Department of Emergency Medicine, Taipei Veterans General Hospital, Taoyuan Branch, Taoyuan 330, Taiwan; 7 Department of Emergency Medicine, Taipei Veterans General Hospital, Taipei 11217, Taiwan; 8 Department of Emergency Medicine, Chung Shan Medical University Hospital, Taichung 40201, Taiwan; 9 Lung Cancer Research Center, Chung Shan Medical University Hospital, Taichung 40201, Taiwan

**Keywords:** *Aeromonas*, bacteremia, mortality risk, scoring systems, temperature

## Abstract

Background: *Aeromonas* species, Gram-negative, non-sporulating, facultative, and anaerobic bacilli, widely distributed in aquatic environments, derive various infections, including bacteremia. Most of these infections were opportunistic and found in patients with predisposing conditions. Among the infections, bacteremia remains with notable mortality, reported from 15% to 45%. However, predicting systems for assessing the mortality risk of this disease have yet to be investigated. We aimed to validate the performance of specific predictive scoring systems to assess the clinical outcomes of *Aeromonas* bacteremia and applied the revised systems to predict mortality risk. Methods: A retrospective observational study reviewed patients with bacteremia caused by *Aeromonas* spp. based on at least one positive blood culture sample collected in the emergency department from January 2012 to December 2020. The outcome was in-hospital mortality. We used seven predictive scoring systems to predict the clinical outcome. According to the effectiveness in predicting mortality, we revised three of the seven predictive scoring systems by specific characteristics to refine their risk-predicting performances. Results: We enrolled 165 patients with bacteremia caused by *Aeromonas* spp., including 121 males (73.3%) and 44 females (26.7%), with a mean age of 66.1 ± 14.9 years and an average length of hospital stay of 12.4 ± 10.9 days. The overall mortality rate was 32.7% (54/165). The non-survivors had significantly higher scores in MEDS (6.7 ± 4.2 vs. 12.2 ± 3.3, *p* < 0.001), NEWS (4.0 ± 2.8 vs. 5.3 ± 3.0, *p* = 0.008), and qSOFA (0.3 ± 0.6 vs. 0.6 ± 0.7, *p* = 0.007). Regarding mortality risk prediction, the MEDS demonstrated the best predictive power with AUC of ROC measured up to 0.834, followed by NEWS (0.626) and qSOFA (0.608). We revised the MEDS, NEWS, and qSOFA by hemoglobin and lactate. We found that the revised scores had better powerful performance, including 0.859, 0.767, and 0.691 of the AUC of ROC, if the revised MEDS ≥10, revised NEWS ≥8, and revised qSOFA ≥2, respectively. Conclusions: MEDS, NEWS, and qSOFA were good tools for predicting outcomes in patients with *Aeromonas* spp. bacteremia. The revised MEDS, NEWS, and qSOFA demonstrated more powerful predicting performance than the original scoring systems. We suggested that patients with higher scores in revised MEDS (≥10), revised NEWS (≥8), and revised qSOFA (≥2) received early goal-directed therapy and appropriate broad-spectrum antibiotic treatment as early as possible to reduce mortality.

## 1. Introduction

*Aeromonas* species are Gram-negative, non-sporulating, facultative, anaerobic tiny bacilli widely distributed in aquatic environments, including lakes, rivers, groundwater, water treatment systems, sewage, and ground soil [1,2,3]. Opportunistic infection from *Aeromonas* species involves various types, including pneumonia, gastrointestinal tract infection, peritonitis, urinary–genital tract infection, soft tissue infection, and bloodstream infection (BSI) [4,5,6,7,8,9,10]. The most reported *Aeromonas* spp. leading to human infection are *A. hydrophila*, *A. caviae*, and *A. sobria* [1,11,12,13].

*Aeromonas* spp. prevalent regions are located primarily in tropical and subtropical areas, and there is an affinity between the prevalence of *Aeromonas* spp. infection and warmer outdoor temperatures [1,14,15]. According to a previous observational study, the average annual incidences of bacteremia due to *Aeromonas* spp. was 76 cases/million inhabitants between 2008 and 2010 in Tainan City, located in southern Taiwan, which discloses higher prevalence than those in the Western countries, ranging from 0.66 to 1.5 cases/million population [16].

Although human infection of *Aeromonas* could occur in healthy people, literature reported most infections in patients with predisposing conditions, including liver disease, malignancy, and immunocompromised conditions [1,13,17,18]. Among the infective diseases derived from *Aeromonas* spp., bacteremia remains a common infection type, with a notable mortality rate as high as 68% reported in immunocompromised patients [14,18,19].

In the emergency department (ED), bacteremia is a life-threatening critical condition, with patients confronting this situation needing emergent management in the ED, including fluid resuscitation, broad-spectrum antibiotics administration, infection source control, and close vital sign monitoring [20]. Because of the high mortality rate, many studies have developed various predictive scoring systems to predict the mortality risk of BSI in the ED [21,22]. Various predictive scoring systems revealed their effectiveness under different situations, such as infectious disease, hospital admission, and clinical management guidance. For example, a recent study applied the Mortality in Emergency Department Sepsis (MEDS) to predict prognosis in patients with community-acquired bacteremia in the ED [23].

Reviewing previous literature, we found scoring systems for predicting the prognosis of bacteremia caused by *Aeromonas* spp. which we scarcely investigated. In this study, we intended to validate the performance of specific clinically available scoring systems (n = 7) to assess this disease’s severity and clinical outcomes and revise scoring systems with several clinical characteristics accordingly. Compared with the original ones, we applied these revised scoring systems to predict the mortality risk in bacteremia caused by *Aeromonas* spp. Furthermore, we describe the epidemiology, especially the case distribution co-relating to seasonal and monthly temperature outdoors, and the clinical characteristics of bacteremia caused by *Aeromonas* spp. in patients who sought treatment at our hospital in central Taiwan. 

## 2. Materials and Methods

### 2.1. Data Collection and Definition

We conducted this retrospective observational study in a tertiary care center in Taiwan (Taichung Veterans General Hospital, TCVGH) that receives ~65,000 ED visits yearly. This study targeted patients with bacteremia of *Aeromonas* spp., approved by the institutional review board of TCVGH (No. CE22240B). Bacteremia of *Aeromonas* spp. was confirmed based on at least one positive blood culture sample collected in the ED. We obtained the data in this study from the electronic clinical database of TCVGH from January 2012 to December 2020. 

Patient data included demographics, laboratory investigations, in-hospital medical intervention, and clinical outcomes. The primary outcome was the in-hospital mortality. We applied univariate and multivariate analyses to evaluate the mortality risk as well. We obtained the average monthly and seasonal temperatures from the Taiwan Central Weather Administration.

### 2.2. Scoring Systems

Given clinical outcomes, we used seven scoring systems we analyzed below: MEDS, Modified Early Warning Score (MEWS), National Early Warning Score (NEWS), Rapid Acute Physiology Score (RAPS), Rapid Emergency Medicine Score (REMS), quick Sequential Organ Failure Assessment (qSOFA), and Worthing Physiological Scoring system (WPS). We revealed indicators in the abovementioned scoring systems, as shown in Table S1 (Supplementary Materials).

### 2.3. Statistic Analysis

We expressed continuous data as mean ± standard deviation (SD) and categorical data as number and percentage. We applied Chi-squared tests to compare categorical data and Mann–Whitney–Wilcoxon U tests to compare continuous data regarding mortality risks in the survivors and non-survivors. We performed univariate and multivariate analyses using the Cox regression model to assess possible predictors for mortality, and we presented the hazard ratio and confidence interval. We used the area under the curve (AUC) of the receiver operating characteristic curve (ROC) to compare predictive power across different scoring systems. We used cut-off points to stratify mortality risks regarding the sensitivity, specificity, negative predictive value (NPV), and positive predictive value (PPV). The *p* values < 0.05 were considered statistically significant. Analyses were performed on the Statistical Package for the Social Science (IBM SPSS version 22.0; International Business Machines Corp., New York, NY, USA) and R (Version 4.1.3, R Foundation for Statistical Computing, Vienna, Austria).

## 3. Results

### 3.1. Demographics, Clinical Characteristics, Primary Outcome, and Comorbidities

We confirmed one hundred sixty-five patients diagnosed with bacteremia caused by *Aeromonas* spp. in the ED. We demonstrated the demographics, clinical characteristics, comorbidities, and primary outcome of 165 patients with bacteremia caused by *Aeromonas* spp. (Table 1). The average annual incidence was 6.6 cases/million population in Taichung City. There were 121 males (73.3%) and 44 females (26.7%), with a mean age of 66.1 ± 14.9 years and an average length of hospital stay (LOS)was 12.4 ± 10.9 days.

The systolic blood pressure (SBP), diastolic blood pressure (DBP), and mean blood pressure (MAP), working as an essential sign of tissue perfusion in sepsis, demonstrated significant differences between the survivors and non-survivors (124.3 ± 26.3 vs. 108.9 ± 23.2, *p* < 0.001, 71.4 ± 13.7 vs. 64.8 ± 18.8, *p* = 0.001, and 87.9 ± 18.3 vs. 79.5 ± 18.9, *p* < 0.001). We found a lower Glasgow Coma Scale (GCS) score in the non-survivors, which meant more patients presenting with depressed consciousness when arriving at ED (14.1 ± 2.5 vs. 9.30 ± 3.7, *p* = 0.019). Fever, considered a signature of infection, presented in both the survivors and non-survivors, with an average body temperature (BT) of 37.7 ± 1.1 °C. However, there was no significance of the BT between the two groups. More oxygen supply (47.8% vs. 66.7%, *p* = 0.034), respiratory failure (0.9% vs. 20.4%, *p* < 0.001), and vasopressor administration (18.0% vs. 51.9%, *p* < 0.001) were observed in the non-survivors. Regarding scoring systems, the non-survivors had significantly higher scores in the MEDS (6.7 ± 4.2 vs.12.2 ± 3.3, *p* < 0.001), the MEWS (1.7 ± 1.9 vs. 2.3 ± 2.0, *p* = 0.04), the NEWS (4.0 ± 2.8 vs. 5.3 ± 3.0, *p* = 0.008), and the qSOFA (0.3 ± 0.6 vs. 0.6 ± 0.7, *p* = 0.007).

Of all 165 patients, 54 died, with a mortality rate of 32.7%. Biliary tract diseases (69.1%), hypertension (62.4%), and malignancy (60.0%) were the three most common comorbidities (Table 1). Other comorbidities included gastrointestinal (GI) diseases (45.5%), esophageal variceal (EV)/gastric variceal (GV) bleeding (40.0%), cirrhosis (29.7%), diabetes mellitus (DM) (26.7%), and end-stage renal disease (ESRD) (25.5%). Interestingly, we found that biliary tract diseases, as comorbidities, were associated with more survivors. On the other hand, malignancy, cirrhosis, EV/GV bleeding, DM, and GI diseases were prone to show in the non-survivors.

### 3.2. Laboratory Data

We presented laboratory data in Table 2. The survivors showed significant differences of white blood cell (WBC) (10,660.9 ± 5780.3 vs. 10,824.6 ± 14,327.1, *p* = 0.04), segmented neutrophil (85.8 ± 10.6 vs. 71.7 ± 27.6, *p* = 0.001), hemoglobin (Hgb) (12.3 ± 2.3 vs. 10.6 ± 2.6, *p* < 0.001), platelet (158.0 ± 91.4 vs. 124.3 ± 92.6, *p* = 0.016), albumin (3.27 ± 0.71 vs. 2.91 ± 0.72, *p* = 0.008), sodium (135.2 ± 4.4 vs. 132.8 ± 7.4, *p* = 0.005), alkaline phosphatase (ALK-P) (229.7 ± 196.7 vs. 337.7 ± 277.8, *p* = 0.011), and lactate (32.2 ± 34.2 vs. 45.2 ± 27.6, *p* < 0.001) than those in the non-survivors.

### 3.3. Microorganisms of Aeromonas *spp.*

All patients in this study received at least one blood sample in the ED, with at least one sample showing positive results for *Aeromonas* spp. Six patients had infected two species of *Aeromonas* spp. at the same time. *A. sobria* (67 isolated, 40.6%), *A. caviae* (50 isolated, 30.3%), *A. hydrophila* (49 isolated, 29.7%), *A. veronii* (3 isolated, 1.8%), and others (2 isolated, 1.2%, including one of *Aeromonas jandaei* and one of unspecific *Aeromonas* spp.), which were isolated from 171 blood samples (Table 3). Notably, bacteremia caused by *A. caviae* led to more survival cases than other species. 

### 3.4. Trend Association of the Case Distribution and the Seasonal and Monthly Temperature

**Figure 1:**
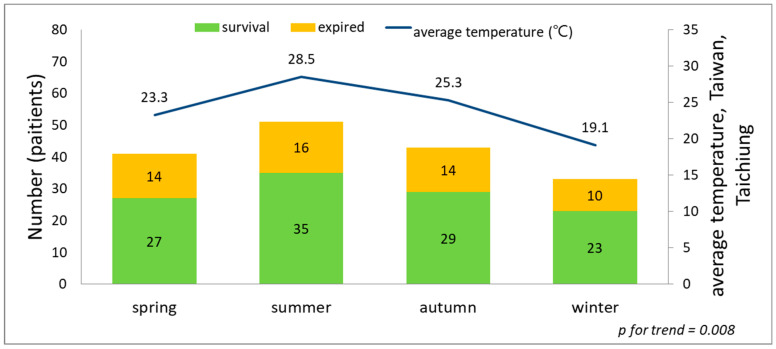
The trend association between the seasonal average temperature and case numbers of bacteremia caused by *Aeromonas* spp., *p* = 0.008.

**Figure 2:**
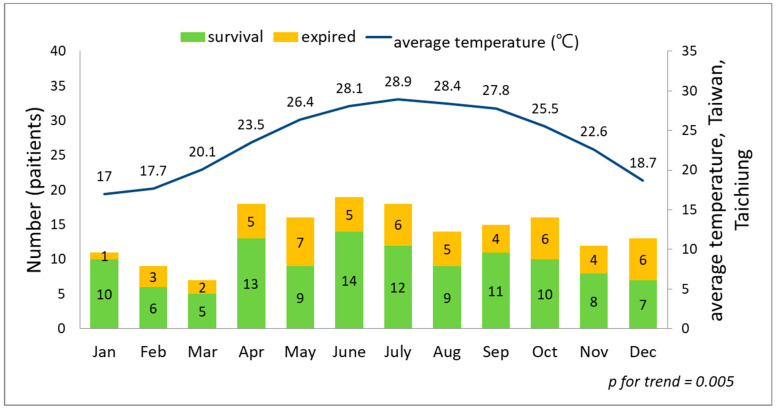
The trend association between the monthly average temperature and case numbers of bacteremia caused by *Aeromonas* spp., *p* = 0.005.

We used linear regression analysis to assess the trends. Both trends showed affinity between the higher average temperature and more case numbers. Figure 1 and Figure 2 depicted a significant trend association between the case numbers and the seasonal and monthly temperatures (*p* = 0.008 and *p* = 0.005). 

### 3.5. Clinical Courses, Management, and Scoring Systems

We observed more oxygen supply (47.8% vs. 66.7%, *p* = 0.034), respiratory failure (0.9% vs. 20.4%, *p* < 0.001), and vasopressor administration (18.0% vs. 51.9%, *p* < 0.001) in the non-survivors. Regarding scoring systems, the non-survivors had significantly higher scores in the MEDS (6.7 ± 4.2 vs.12.2 ± 3.3, *p* < 0.001), the MEWS (1.7 ± 1.9 vs. 2.3 ± 2.0, *p* = 0.04), the NEWS (4.0 ± 2.8 vs. 5.3 ± 3.0, *p* = 0.008), and the qSOFA (0.3 ± 0.6 vs. 0.6 ± 0.7, *p* = 0.007) in Table 4. 

### 3.6. Univariate Analysis of Risk Factors

Table 5 shows the results of univariate analyses for predisposing factors on primary outcome with significant differences between the survivors and non-survivors. We found longer total LOS, intensive care unit (ICU) admission, oxygen supply, respiratory failure, the use of vasopressor, DM, cirrhosis, GI tract diseases, malignancy, hepatitis B, EV/GV bleeding, Hgb, platelet, segmented neutrophil, albumin, sodium, and lactate, which showed significant differences between the survivors and non-survivors. The non-survivors had higher MEDS, NEWS, RAPS, and qSOFA scores than the survivors.

### 3.7. Multivariate Analysis of Risk Factors

Based on the result of univariate analysis, we performed multivariate logistic regression analyses for the MEDS, NEWS, and qSOFA; the scoring systems showed significant differences (Table 6). 

### 3.8. The ROC and Kaplan–Meier Survival Curve of Original and Revised Scoring Systems

**Figure 3:**
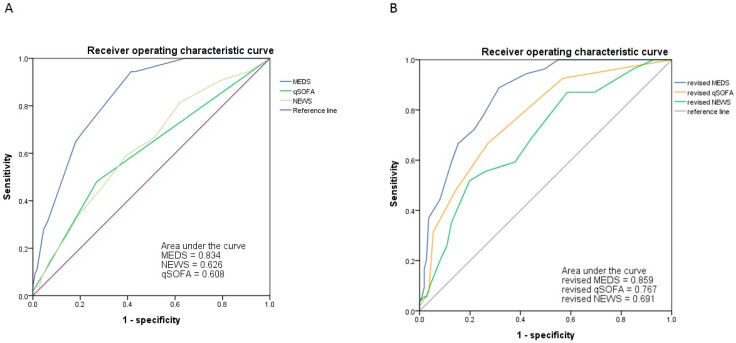
The AUC of ROC for the MEDS, NEWS, and qSOFA indicated 0.834, 0.626, and 0.608 to predict the mortality risks of patients with bacteremia caused by *Aeromonas* spp. (Panel (**A**)). The AUC of ROC for the revised MEDS, NEWS, and qSOFA indicated 0.859, 0.767, and 0.691 to predict the mortality risks of patients with bacteremia caused by *Aeromonas* spp. (Panel (**B**)). AUC, Area under the curve; MEDS, Mortality in Emergency Department Sepsis; NEWS, National Early Warning Score; qSOFA, quick Sepsis Related Organ Failure Assessment; ROC, Receiver operating characteristic curve.

**Figure 4:**
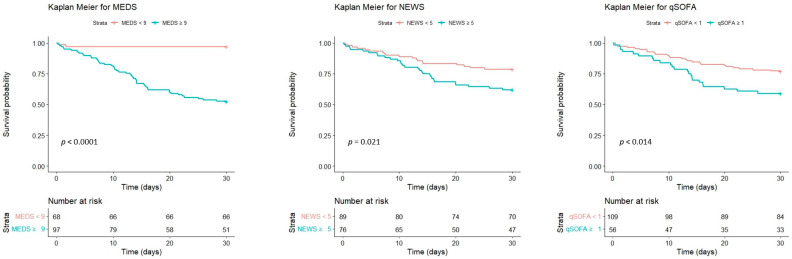
The cumulative survival rates of patients with bacteremia caused by *Aeromonas* spp. were calculated to predict the 30-day mortality rate using Kaplan–Meier analyses. The cut-off point of the MEDS, NEWS, and qSOFA was 9, 5, and 1, respectively. MEDS, Mortality in Emergency Department Sepsis; NEWS, National Early Warning Score; qSOFA, quick Sequential Organ Failure Assessment.

We analyzed the ROC of MEDS, NEWS, and qSOFA regarding accuracy to predict the mortality risk. We depicted the results in Figure 3 and Table 7. The cut-off point of the MEDS was 9, with the area under the curve (AUC) measured up to 0.834 (sensitivity of 94.4% and specificity of 58.6%, *p* < 0.001). The NEWS and qSOFA possessed 5 and 1 as cut-off points, with the AUC of ROC measured up to 0.626 (sensitivity of 53.9% and specificity of 60.4%, *p* = 0.009) and 0.608 (sensitivity of 48.1% and specificity of 73.0%, *p* = 0.025), respectively. We calculated the cumulative survival rates of patients with bacteremia caused by *Aeromonas* spp. using Kaplan–Meier analyses to predict the 30-day mortality rate. The cut-off point of the MEDS, NEWS, and qSOFA was 9, 5, and 1, with significant difference (*p* < 0.0001, *p* = 0.021, and *p* = 0.014), respectively (Figure 4).

**Figure 5:**
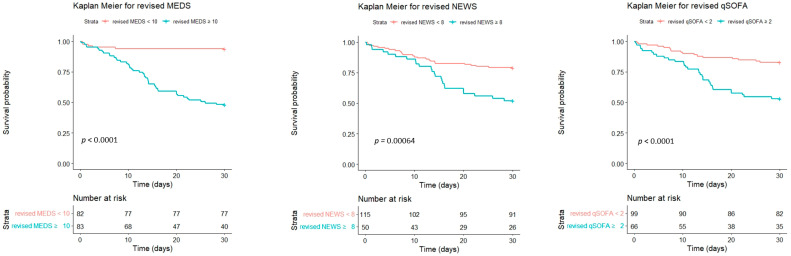
The cumulative survival rates of patients with bacteremia caused by *Aeromonas* spp. were calculated to predict the 30-day mortality rate using Kaplan–Meier analyses. The cut-off point of the revised MEDS, NEWS, and qSOFA was 10, 8, and 2, respectively. MEDS, Mortality in Emergency Department Sepsis; NEWS, National Early Warning Score; qSOFA, quick Sequential Organ Failure Assessment.

We used the Youden index to determine the first and second cut-off points of Hgb and lactate. We assigned each predictor a score of 1 or 2 depending on the HR of the dichotomized variables. Patients obtained additional scores in the MEDS, NEWS, and qSOFA: if Hgb < 12 g/dL, the score was 1; if Hgb < 9 g/dL, the score was 2; if lactate > 37 mg/dL, the score was 1; if lactate > 53mg/dL, the score was 2. We revised the MEDS, NEWS, and qSOFA to refine performance for predicting the mortality risk. Figure 3 and Table 8 depicted the analyses for the ROC of the revised scores. The revised scoring systems’ predictive performance was superior to the original ones, including the revised MEDS (AUC of ROC: 0.859, sensitivity of 88.9%, specificity of 68.5%, *p* < 0.001), revised NEWS (AUC of ROC: 0.767, sensitivity of 66.7, specificity of 73.0%, *p* < 0.001) and revised qSOFA (AUC of ROC: 0.691, sensitivity of 51.9%, specificity of 80.2%, *p* < 0.001). Using Kaplan–Meier analyses, we calculated the cumulative survival rates of patients with bacteremia caused by *Aeromonas* spp. to predict the 30-day mortality rate. The cut-off point of the revised MEDS, NEWS, and qSOFA was 10, 8, and 2, with significant differences (*p* < 0.0001, *p* = 0.00064, and *p* < 0.0001), respectively (Figure 5). The revised scoring systems exhibited more powerful predicting performances than the original ones.

### 3.9. Discriminations Plots

**Figure 6:**
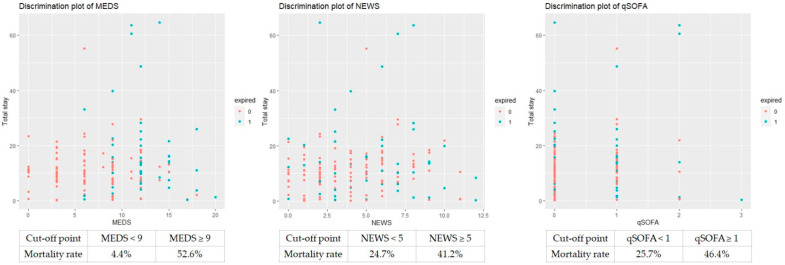
The overall mortality case numbers of the MEDS, NEWS, and qSOFA were 52.6%, 41.2%, and 46.4% if the cut-off point was more than 9, 5, and 1. MEDS, Mortality in Emergency Department Sepsis; NEWS, National Early Warning Score; qSOFA, and quick Sequential Organ Failure Assessment.

**Figure 7:**
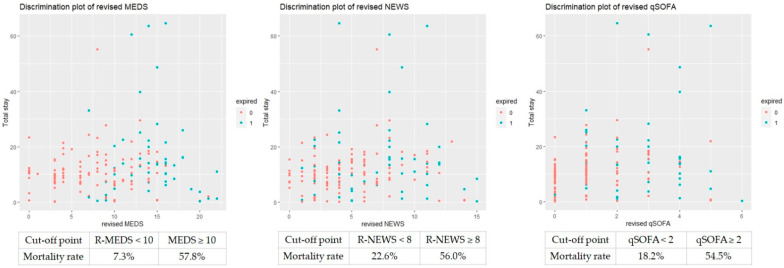
The overall mortality case numbers of the revised MEDS, NEWS, and qSOFA were 57.8%, 56.0%, and 54.5% if the cut-off point was more than 10, 8, and 2. R-MEDS, Revised Mortality in Emergency Department Sepsis; R-NEWS, Revised National Early Warning Score; R-qSOFA, and Revised quick Sequential Organ Failure Assessment.

The discrimination plots showed the overall mortality rate of the MEDS, NEWS, and qSOFA 52.6%, 41.2%, and 46.4% if the cut-off point was more than 9, 5, and 1 (Figure 6). The discrimination plots revealed the overall mortality rate of the revised MEDS, NEWS, and qSOFA 57.8%, 56.0%, and 54.5% if the cut-off point was more than 10, 8, and 2 (Figure 7).

## 4. Discussion

This single-center study investigated 165 patients with bacteremia of *Aeromonas* spp. in a medical center located in central Taiwan within ten years. In Western countries, the bacteremia caused by *Aeromonas* spp. was 0.66 to 1.5 cases/million population. In Tainan, a city in southern Taiwan, the reported incidence was 76/million population from 2008 to 2010 [16]. In our study, the incidence from January 2012 to December 2020 in Taichung, a city located north of Tainan, was 6.6/million population. Earlier literature reported seasonal distribution of infection caused by *Aeromonas* spp., with more frequent occurrence in months with warmer temperatures [1,13,15]. Our study also revealed a significant trend toward more events in warmer months (Figure 1 and Figure 2). This seasonality may be attributed to mesophilic aeromonads growing optimally at warmer water temperatures, causing higher concentrations of bacteria in freshwater environments and domestic water systems [1]. 

The overall mortality rate of bacteremia caused by *Aeromonas* spp. was 15% to 45% [1,13,14,17,24]. Our study showed a mortality rate of 32.7% (54/165), which was compatible with earlier data. The most yielded species were *A. sobria*, *A. caviae*, and *A. hydrophila*. Part of these species correlated with patient outcomes. In our univariate analysis, bacteremia caused by *A. sobria* showed more fatalities, whereas *A. caviae* was associated with favorable results (Table 3). *A. sobria* had been reported as causing more risk in systemic infection or disseminated disease in earlier literature, attributing to its ability to possessing invasive capability in human HEp-2 cells [25]. In vitro studies also demonstrated its heightened pathogenicity and virulence in mice [26]. Our finding could help physicians distinguish high-risk patients by yielding *Aeromonas* species. 

Early research on this disease revealed the most associated comorbidities: hepatobiliary diseases, malignancy, and DM [1,13,14,18,27]. In our study, biliary tract diseases (69.1%), hypertension (62.4%), and malignancy (60.0%), followed by GI tract diseases, EV/GV bleeding, cirrhosis, DM, and ESRD, were the most common comorbidities. Among these diseases, DM, GI tract diseases, malignancies, and liver diseases (including cirrhosis, chronic hepatitis B, and EV/GV bleeding) were risk factors for fatality in our univariate analysis. Immunocompromised status in patients with bacteremia of *Aeromonas* spp. was the crux relating to mortality. The most mentioned conditions in the earlier literature were hepatobiliary diseases, malignancies, DM, ESRD, and diseases in need of immunosuppressants [3,13,14,17]. Interestingly, in our study, biliary tract diseases were prone to shown in survivors, with significantly lower odds ratio (OR) for patients in univariate analysis (OR: 0.40, 95% CI: 0.20–0.80, *p* = 0.010). Studies of biliary sepsis related to *Aeromonas* spp. were most associated with cholelithiasis or choledocholithiasis, cholangiocarcinoma, pancreatic carcinoma, or non-malignant biliary strictures [28,29]. In most instances, the pathological bacteria may originate from the GI tract. With the underlying condition above, more bacteria invade ascending from the GI tract into the biliary system, leading to biliary tract infection, sepsis, and even bacteremia [30,31]. In the ED, early appropriate antibiotic treatment and solution of biliary tract obstruction were our priorities in managing biliary tract infections. The attempt of ED physicians to recognize possible biliary tract infection early ameliorated the outcomes. In a retrospective study reviewing 30 cases of suppurative cholangitis caused by *Aeromonas* spp., with successful drainage of biliary obstruction, infection of *Aeromonas* spp. did not appear to influence the clinical outcome of acute suppurative cholangitis despite the high mortality of biliary sepsis [28]. Under this premise, the better result of bacteremia caused by *Aeromonas* spp. in patients with biliary tract diseases might be attributed to early recognition of possible biliary tract infection, leading to early appropriate management in the ED.

Cirrhotic patients are under systemic multifactorial immune dysfunction status due to multiple pathological mechanisms, leading to substantial mortality or morbidity in bacteremia. Possible pathogenesis included bacterial translocation via the GI tract, portosystemic shunting, allowing more endotoxin in portal circulation to bypass the liver, and dysfunction of phagocyte and opsonic activity with depression of the reticuloendothelial system [15,32,33,34]. Compared with other comorbidities, cirrhosis and cirrhotic-related diseases (HBV carrier and EV/GV bleeding), we had contributed to significantly higher OR of mortality in our study. Variceal bleeding, the most common cause of GI tract bleeding in cirrhotic patients, has been documented with a fatality rate of up to 20% [35]. As cirrhosis progresses in severity, more uncontrolled variceal bleeding appears, with more mortality cases. Outcomes of bacteremia in these patients also worsened with liver decompensated progression [36]. The literature demonstrated bacterial infection in cirrhotic patients as an independent risk factor of uncontrolled GI bleeding, shock, and early mortality [34,37,38]. The hypothesis was based on releasing endotoxin into the systemic circulation, increasing portal pressure through the induction of vasoconstrictive cyclooxygenase products [34]. Notably, our study found EV/GV bleeding contributing to an accentuating risk to outcome in univariate analysis (OR: 3.29, 95% CI: 1.68–6.48, *p* = 0.001) when being a comorbidity of bacteremia caused by *Aeromonas* spp. This result was consistent with the strong association between variceal bleeding and bacterial infection described in the studies. Thus, we advocate patients with bacteremia of *Aeromonas* spp. or suspected cases in the ED presenting with variceal bleeding simultaneously to undergo aggressive monitoring and early hemostatic procedures, with adequate broad-spectrum antibiotics administration. 

Numerous scoring systems are available for predicting and stratifying patient conditions and identifying disease severity and potentially critical situations in the ED. A higher score revealed a higher mortality rate among all seven scoring systems analyzed in our study. The MEDS, NEWS, RAPS, and qSOFA owned significantly higher scores in our univariate analysis. Shapiro et al. developed the MEDS in 2003, and its remarkable performance in predicting mortality in suspected infection or bacteremia in ED has been validated [23,39,40]. The NEWS was first published in 2012, with great ability and high AUROC in predicting patients at risk of cardiac arrest, unplanned ICU admission, or death within 24 h [41,42]. As a rapid screening tool for septic shock, the qSOFA remained a benchmark in rapidly measuring the severity of septic patients. A study from Peru in 2021 showed qSOFA ≥ 2 as a prognostic factor for mortality, focusing on bacteremia of *A. sobria* in 37 patients with hematologic malignancies [43]. The RAPS was abbreviated from the APECHE-II score in 1987 to evaluate critical care transport. Moreover, it was applied to predict infectious disease outcomes in the ED [40]. Of the scoring systems mentioned, the MEDS demonstrated the best prediction performance for the clinical prognosis in our study. The MEDS was calculated based on multiple parameters, including terminal illness, presence of tachypnea or hypoxia, septic shock, platelets count < 150 *×* 10^3^/mm^3^, age > 65 years, lower respiratory infection, nursing home resident, and altered mental state [44]. Our analysis showed the AUC of ROC of MEDS reaching up to 0.834, with a sensitivity of 94.4% and a specificity of 58.6%, under a cut-off point 9. To our knowledge, none of the literature has focused on this excellent performance of outcome prediction in such a high-morality disease. The availability of the MEDS as a predictive tool could benefit ED and ICU physicians confronting this disease. The NEWS and qSOFA also showed acceptable performances in predicting the mortality risk, with the AUC of ROC 0.626 and 0.608, respectively (Figure 3). 

In the univariate analysis of laboratory data, we found mortality associated with higher alkaline phosphatase and lactate levels, lower hemoglobin levels, platelet count, segmented neutrophil level, albumin, and sodium (Table 5). Since we determined to refine the models of the abovementioned scoring systems for better predictive performances, we conducted revisions of them with certain laboratory variables. Anemia was known as one of the most common complications in the clinical course of sepsis, and its relationship with mortality of sepsis had been reported in earlier literature [45]. Lactic acid level has been widely used to predict sepsis progression and disease prognosis [20]. Neither laboratory variable (Hbg and lactate) indicated the original MEDS, NEWS, and qSOFA. Based on our univariate analysis, we assigned each scoring system with additional scores by Hgb level (Hgb < 12 g/dL, score = 1; Hgb < 9 g/dL, score = 2) and lactate (lactate > 37 mg/dL, score = 1; lactate > 53 mg/dL, score = 2). The analyses for accuracy in predicting the mortality of revised models (documented as the revised MEDS, revised NEWS, and revised qSOFA) demonstrated excellent discrimination compared with the original models (Figure 6 and Figure 7). The revised MEDS remained a perfect tool for predicting mortality, with the AUC of the ROC reaching 0.859. The high sensitivity, up to 88.9%, could allow ED physicians to narrow in patients requiring emergent management. The revised NEWS and qSOFA also demonstrated that the AUC of ROC exceeded the original models, with the AUC of the ROC of 0.767 and 0.691, respectively. Both revised models showed less effectiveness in predicting mortality than the revised MEDS. However, we still advocated them as valuable tools in predicting high-risk patients. The NEWS included respiratory rate, oxygen saturation, necessity of oxygen supply, BT, SBP, heart rate, and consciousness level [42], the variables routinely measured during the ED observation. The qSOFA was even more simplified, with variables including altered mental status (GCS < 15), respiratory rate ≥ 22/min, and systolic BP ≤ 100mmHg [20]. With the easily accessible parameters that the NEWS and qSOFA incorporated, we believed both revised models could still facilitate early recognition of high-risk patients for ED and ICU physicians.

## 5. Limitations

There are several limitations in the study. First, although we enrolled many cases with bacteremia of *Aeromonas* spp., this is a single-center study with a retrospective nature. Thus, data and clinical variables involved in this study might not represent the complete characteristics of this disease. Clinical symptoms and disease progression were investigated retrospectively, possibly resulting in inevitable bias. Second, as the primary treatment for bacteremia, antibiotics were the key to adequate management. We did not record the detailed antibiotics information. Therefore, we could not analyze and compare the clinical outcomes according to those treatments. Third, patients in this study mostly had multiple comorbidities, so co-infection with other bacteria spices or co-infection of more than one spice of *Aeromonas* spp. was not uncommon. It might cause information bias in disease prognosis or outcomes. Fourth, owing to the rarity of bacteremia caused by *Aeromonas* spp. and inoculation time for bacteria growth in blood samples, it was complex to confirm and identify the diagnosis in the ED but also challenging to conduct a prospective study.

## 6. Conclusions

Bacteremia of *Aeromonas* spp. was a disease with a high mortality rate. Physicians should be highly aware of patients suspected of this disease, especially those with DM, liver diseases (including cirrhosis, hepatitis B carrier, and GV/EV bleeding), GI tract diseases, and malignancy. The MEDS, NEWS, RAPS, and qSOFA effectively predicted the mortality risk, with the MEDS (≥9) performing the best. The revised MEDS, NEWS, and qSOFA systems demonstrated excellent discrimination compared with the original models by modifying the hemoglobin and serum lactate scoring system. The revised MEDS (≥10) still best-predicted mortality risk. The revised NEWS and qSOFA facilitated fair predicting performances and were good choices for physicians due to their accessibility. Further large-scale studies are necessary to provide more accurate clinical guidance, risk prediction, and detailed disease progression. 

## Figures and Tables

**Table 1:** Demographics, clinical characteristics, comorbidities, and primary outcome of 165 patients with bacteremia caused by *Aeromonas* spp.

General Data	All (*n* = 165)	Survival (*n* = 111)	Expired (*n* = 54)	*p*-Value
Sex				0.736
Male	121 (73.3%)	80 (72.1%)	41 (75.9%)	
Female	44 (26.7%)	31 (27.9%)	13 (24.1%)	
Age	66.1 ± 14.9	63.8 ± 14.7	64.4 ± 15.3	0.298
Vital Signs				
SBP	118.6 ± 26.2	123.4 ± 26.3	108.9 ± 23.2	<0.001 **
DBP	69.2 ± 15.8	71.4 ± 13.7	64.8 ± 18.8	0.001 **
MAP	85.2 ± 18.9	87.9 ± 18.3	79.5 ± 18.9	<0.001 **
HR	102.0 ± 20.1	101.7 ± 21.5	102.6 ± 17.0	0.484
RR	18.8 ± 2.3	18.7 ± 2.1	18.9 ± 2.5	0.869
BT	37.7 ± 1.1	37.8 ± 1.2	37.71 ± 1.1	0.917
GCS	12.1 ± 3.8	14.1 ± 2.5	9.30 ± 3.7	0.019 *
SpO_2_	95.4 ± 8.6	95.0 ± 10.2	96.4 ± 3.2	0.360
Comorbidities				
CVA	10 (6.1%)	9 (8.1%)	1 (1.9%)	0.168
CHF	16 (9.7%)	10 (9.0%)	6 (11.1%)	0.882
DM	44 (26.7%)	23 (20.7%)	21 (38.9%)	0.022 *
Hypertension	103 (62.4%)	70 (63.1%)	33 (61.1%)	0.943
Biliary tract diseases	114 (69.1%)	84 (75.7%)	30 (55.6%)	0.014 *
GI diseases	75 (45.5%)	43 (38.7%)	32 (59.3%)	0.020 *
ESRD	42 (25.5%)	25 (22.5%)	17 (31.5%)	0.294
Post-transplant status	5 (3.0%)	3 (2.7%)	2 (3.7%)	0.663
Autoimmune disorder	6 (3.6%)	2 (1.8%)	4 (7.4%)	0.090
Malignancy	99 (60.0%)	60 (54.1%)	39 (72.2%)	0.039 *
Liver diseases				
HCC	25 (15.2%)	15 (13.5%)	10 (18.5%)	0.542
HBV carrier	19 (11.5%)	8 (7.2%)	11 (20.4%)	0.026 *
HCV carrier	6 (3.6%)	3 (2.7%)	3 (5.6%)	0.394
Alcoholism	27 (16.4%)	16 (14.4%)	11 (20.4%)	0.456
EV/GV bleeding	66 (40.0%)	34 (30.6%)	32 (59.3%)	0.001 **
Cirrhosis	49 (29.7%)	27 (24.3%)	22 (40.7%)	0.047 *

Chi–squared test. Mann–Whitney U-test. * *p* < 0.05, ** *p* < 0.01, Statistically significant. Continuous data were expressed as mean ± SD. Categorical data were expressed as number and percentage. BT, Body temperature; CHF, Chronic heart failure; CVA, Cerebrovascular accident; DBP, Diastolic blood pressure; DM, Diabetes mellitus; ESRD, End-stage renal disease; EV, Esophageal varices; GCS, Glasgow coma scale; GI, Gastrointestinal; GV, Gastric varices; HBV, hepatitis B virus; HCC, Hepatocellular carcinoma; HCV, hepatitis C virus; HR, Heart rate; MAP, Mean blood pressure; RR, Respiratory rate; SBP, Systolic blood pressure.

**Table 2:** Laboratory data of 165 patients with bacteremia caused by *Aeromonas* spp.

Variables	All (*n* = 165)	Survival (*n* = 111)	Expired (*n* = 54)	*p*-Value
Complete blood cells				
WBC	10714.5 ± 9420.9	10660.9 ± 5780.3	10824.6 ± 14327.1	0.040 *
Hgb	11.8 ± 2.6	12.3 ± 2.3	10.6 ± 2.6	<0.001 **
Platelet	147.0 ± 92.9	158.0 ± 91.4	124.3 ± 92.6	0.016 *
Segmented Neutrophil	81.2 ± 19.1	85.8 ± 10.6	71.7 ± 27.6	0.001 **
Biochemistry				
Albumin	3.13 ± 0.73	3.27 ± 0.71	2.91 ± 0.72	0.008 **
Total bilirubin	4.25 ± 5.19	4.32 ± 5.85	4.10 ± 3.53	0.291
ALK-P	264.5 ± 230.8	229.7 ± 196.7	337.7 ± 277.8	0.011 *
GPT	115.8 ± 142.5	116.4 ± 139.3	114.5 ± 150.3	0.666
Creatinine	1.39 ± 1.42	1.42 ± 1.59	1.32 ± 1.01	0.748
Sodium	134.4 ± 5.6	135.2 ± 4.4	132.8 ± 7.4	0.005 **
CRP	6.27 ± 7.08	5.89 ± 7.08	7.03 ± 7.09	0.057
Lactate	36.6 ± 32.6	32.2 ± 34.2	45.2 ± 27.6	<0.001 **
Glucose	148.8 ± 62.6	145.4 ± 61.3	155.6 ± 65.5	0.431
Coagulation Function				
PT	13.47 ± 4.53	13.25 ± 5.00	13.90 ± 3.49	0.002 **
aPTT	33.44 ± 11.75	33.03 ± 13.05	34.24 ± 8.77	0.042 *
Arterial Blood Gas				
pH	7.39 ± 0.09	7.38 ± 0.09	7.41 ± 0.09	0.257

Chi–squared test. Mann–Whitney U-test. * *p* < 0.05, ** *p* < 0.01, Statistically significant. Continuous data were expressed as mean ± SD. ALK-P, Alkaline phosphatase; aPTT, activated partial thromboplastin time; CRP, C-reactive protein; GPT, Glutamic pyruvic transaminase; Hgb, Hemoglobin; PT, Prothrombin time; WBC, White blood cell.

**Table 3:** Microorganisms of 165 patients with bacteremia caused by *Aeromonas* species.

Microorganisms	All (*n* = 165)	Survival (*n* = 111)	Expired (*n* = 54)	*p*-Value
*A. hydrophila*	49 (29.7%)	30 (27.0%)	19 (35.2%)	0.371
*A. sobria*	67 (40.6%)	39 (35.1%)	28 (51.9%)	0.060
*A. caviae*	50 (30.3%)	43 (38.7%)	7 (13.0%)	0.001 **
*A. veronii* ^f^	3 (1.8%)	2 (1.8%)	1 (1.9%)	1.000
Others ^f,#^	2 (1.2%)	2 (1.8%)	0 (0.0%)	1.000

Chi–Square test. ^f^ Fisher’s exact test. ** *p* < 0.01, Statistically significant. Categorical data were expressed as numbers and percentages. ^#^ *Aeromonas jandaei* and unspecific *Aeromonas* spp.

**Table 4:** Clinical courses, management, and scoring systems of 165 patients with bacteremia caused by *Aeromonas* spp.

General Data	All (*n* = 165)	Survival (*n* = 111)	Expired (*n* = 54)	*p*-Value
Clinical Courses				
ICU admission	21 (12.7%)	6 (5.4%)	15 (27.8%)	<0.001 **
Respiratory failure	12 (7.3%)	1 (0.9%)	11 (20.4%)	<0.001 **
Total stay (day)	12.4 ± 10.9	10.8 ± 7.5	15.9 ± 15.2	0.078
ICU stay (day)	14.1 ± 15.4	7.83 ± 2.9	16.60 ± 17.6	0.381
Management				
Oxygen supply	89 (53.9%)	53 (47.8%)	36 (66.7%)	0.034 *
Intubation	12 (7.3%)	1 (0.9%)	11 (20.4%)	<0.001 **
Septic shock ^f^	7 (4.2%)	3 (2.7%)	4 (7.4%)	0.218
Vasopressor use	48 (29.1%)	20 (18.0%)	28 (51.9%)	<0.001 **
Scoring Systems				
MEDS	8.5 ± 4.7	6.7 ± 4.2	12.2 ± 3.3	<0.001 **
MEWS	1.9 ± 1.9	1.7 ± 1.9	2.3 ± 2.0	0.040 *
NEWS	4.4 ± 2.9	4.0 ± 2.8	5.3 ± 3.0	0.008 **
RAPS	1.4 ± 1.5	1.2 ± 1.4	1.7 ± 1.7	0.065
REMS	5.6 ± 2.5	5.6 ± 2.5	5.7 ± 2.7	0.946
qSOFA	0.4 ± 0.6	0.3 ± 0.6	0.6 ± 0.7	0.007 **
WPS	2.1 ± 1.8	1.9 ± 1.8	2.5 ± 1.9	0.037 *

Chi–squared test. ^f^ Fisher’s Exact test. Mann–Whitney U-test. * *p* < 0.05, ** *p* < 0.01, Statistically significant. ICU, Intensive care unit ICU, Intensive care unit; MEDS, Mortality in Emergency Department Sepsis; MEWS, Modified Early Warning Score; NEWS, National Early Warning Score; RAPS, Rapid Acute Physiology Score; REMS, Rapid Emergency Medicine Score; qSOFA, quick Sequential Organ Failure Assessment; WPS, Worthing Physiological Scoring system.

**Table 5:** Univariate logistic analysis for 165 patients with bacteremia caused by *Aeromonas* species.

Characteristics	Odds Ratios	95% Confidence Interval	*p*-Value
SBP	0.98	0.96–0.99	0.001 **
DBP	0.97	0.95–0.99	0.013 *
MAP	0.97	0.96–0.99	0.009 **
GCS	0.62	0.41–0.94	0.026 *
Clinical course			
Total hospital stay (day)	1.044	1.01–1.08	0.010 *
ICU admission (day)	6.73	2.44–18.58	<0.001 **
Intubation	28.14	3.53–224.62	0.002 **
Clinical Management			
Oxygen supply	2.19	1.11–4.31	0.023 *
Intubation	28.14	3.53–224.62	0.002 **
Vasopressor use	4.90	2.38–10.07	<0.001 **
Comorbidities			
DM	2.44	1.19–4.97	0.015 *
Biliary tract disease	0.40	0.20–0.80	0.010 *
GI tract disease	2.30	1.18–4.47	0.014 *
Malignancy	2.21	1.09–4.46	0.027 *
Cirrhosis	2.14	1.07–4.29	0.032 *
Hepatitis B carrier	3.29	1.24–8.76	0.017 *
EV/GV bleeding	3.29	1.68–6.48	0.001 **
Laboratory data			
Hgb	0.76	0.65–0.87	<0.001 **
Platelet	1.00	0.99–1.00	0.031 *
Segmentedneutrophil	0.96	0.94–0.98	<0.001 **
Albumin	0.50	0.29–0.85	0.010 *
ALK-P	1.00	1.00–1.00	0.012 *
Sodium	0.92	0.87–0.98	0.012 *
Lactate	1.01	1.00–1.02	0.035 *
Scoring systems			
MEDS	1.42	1.26–1.59	<0.001 **
NEWS	1.16	1.04–1.30	0.010 *
RAPS	1.25	1.01–1.54	0.041 *
qSOFA	2.00	1.19–3.36	0.009 **

Logistic regression analysis. * *p* < 0.05, ** *p* < 0.01, Statistically significant. ALK-P, Alkaline phosphatase; DBP, Diastolic blood pressure; DM, Diabetes Mellitus; EV/GV, Esophageal varices/Gastric varices; GCS, Glasgow coma scale; GI, Gastrointestinal; MAP, Mean blood pressure; MEDS, Mortality in Emergency Department Sepsis; NEWS, National Early Warning Score; RAPS, Rapid Acute Physiology Score; SBP, Systolic blood pressure; qSOFA, quick Sequential Organ Failure Assessment.

**Table 6:** Univariate and multivariate logistic regression analyses for primary outcome.

Scoring Systems	Univariate			Multivariate		
		95% CI	*p*-Value	OR	95%	*p*-Value
MEDS	1.42	1.26–1.59	<0.001 **	1.53	1.32–1.76	<0.001 **
NEWS	1.16	1.04–1.30	0.010 *	0.86	0.70–1.04	0.118
qSOFA	2.00	1.19–3.36	0.009 **	1.29	0.56–2.96	0.550

* *p* < 0.05, ** *p* < 0.01, Statistically significant. Abbreviations: CI, Confidence interval; MEDS, Mortality in Emergency Department Sepsis; NEWS, National Early Warning Score; qSOFA, quick Sequential Organ Failure Assessment; OR, Odds ratios.

**Table 7:** The AUC of ROC, cut-off point (COP), sensitivity, specificity, positive predictive value (PPV), negative predictive value (NPV), accuracy, and standard error (SE) to predict the mortality risk by the MEDS, NEWS, and qSOFA.

Scores	AUC	COP	Sensitivity	Specificity	PPV	NPV	Accuracy	SE	*p*-Value
MEDS	0.834	9	94.4%	58.6%	52.6%	95.6%	70.3%	0.031	<0.001 **
NEWS	0.626	5	59.3%	60.4%	42.1%	75.3%	60.0%	0.046	0.009 **
qSOFA	0.608	1	48.1%	73.0%	46.4%	74.3%	64.8%	0.048	0.025 *

* *p* < 0.05, ** *p* < 0.01, Statistically significant; Abbreviations: AUC, Area under the curve; MEDS, Mortality in Emergency Department Sepsis; NEWS, National Early Warning Score; qSOFA, quick Sequential Organ Failure Assessment; NPV, negative predictive value; PPV, positive predictive value; ROC, Receiver operating characteristic curve; SE, Standard error.

**Table 8:** The AUC of ROC, cut-off point (COP), sensitivity, specificity, positive predictive value (PPV), negative predictive value (NPV), accuracy, and standard error (SE) to predict the mortality risk by the revised MEDS, revised qSOFA and revised NEWS.

Scores	AUC	COP	Sensitivity	Specificity	PPV	NPV	Accuracy	SE	*p*-Value
R–MEDS	0.859	10	88.9%	68.5%	57.8%	92.7%	75.2%	0.028	<0.001 **
R–qSOFA	0.767	2	66.7%	73.0%	54.5%	81.8%	70.9%	0.038	<0.001 **
R–NEWS	0.691	8	51.9%	80.2%	56.0%	77.4%	70.9%	0.043	<0.001 **

** *p* < 0.01, Statistically significant; Abbreviations: AUC, Area under the curve; R-MEDS, Revised Mortality in Emergency Department Sepsis; R-NEWS, Revised National Early Warning Score; R-qSOFA, Revised quick Sequential Organ Failure Assessment; NPV, negative predictive value; PPV, positive predictive value; ROC, Receiver operating characteristic curve; SE, Standard error.

## Data Availability

Readers can access the data and material supporting the study’s conclusions by contacting Sung-Yuan Hu at song9168@pie.com.tw.

## Supplementary Materials

The following supporting information can be downloaded at: https://www.mdpi.com/article/10.3390/diagnostics14020124/s1, Table S1: Scoring systems [46,,47,48,49].

## References

[1] Janda J.M., Abbott S.L. (2010). The genus *Aeromonas*: Taxonomy, pathogenicity, and infection. Clin. Microbiol. Rev..

[2] Parker J.L., Shaw J.G. (2011). *Aeromonas* spp. Clinical microbiology and disease. J. Infect..

[3] Tang H.J., Lai C.C., Lin H.L., Chao C.M. (2014). Clinical manifestations of bacteremia caused by *Aeromonas* species in southern Taiwan. PLoS ONE.

[4] Katz M.J., Parrish N.M., Belani A., Shah M. (2015). Recurrent *Aeromonas* Bacteremia Due to Contaminated Well Water. Open Forum Infect. Dis..

[5] Janda J.M., Abbott S.L. (1998). Evolving Concepts Regarding the Genus *Aeromonas*: An Expanding Panorama of Species, Disease Presentations, and Unanswered Questions. Clin. Infect. Dis..

[6] Janda J.M. (1991). Recent Advances in the Study of the Taxonomy, Pathogenicity, and Infectious Syndromes Associated with the Genus *Aeromonas*. Clin. Microbiol. Rev..

[7] Bauah F.K., Ahmed N.H., Grover R.K. (2015). Surgical Site Infection Caused by *Aeromonas hydrophila* in a Patient with Underlying Malignancy. J. Clin. Diagn. Res. JCDR.

[8] Gold W.L., Salit I.E. (1993). *Aeromonas hydrophila* Infections of Skin and Soft Tissue: Report of 11 Cases and Review. Clin. Infect. Dis..

[9] Lamy B., Kodjo A., Laurent F. (2009). Prospective nationwide study of *Aeromonas* infections in France. J. Clin. Microbiol..

[10] Xu C., Lin Q., Zhao Y., Zhu G., Jiang E., Li S., Mi Y., Zheng Y., Zhang F., Zhu X. (2022). Clinical characteristics and risk factors of *Aeromonas* bloodstream infections in patients with hematological diseases. BMC Infect. Dis..

[11] Chuang H.C., Ho Y.H., Lay C.J., Wang L.S., Tsai Y.S., Tsai C.C. (2011). Different clinical characteristics among *Aeromonas hydrophila*, *Aeromonas veronii biovar sobria* and *Aeromonas caviae* monomicrobial bacteremia. J. Korean Med. Sci..

[12] Wu C.J., Chen P.L., Hsueh P.R., Chang M.C., Tsai P.J., Shih H.I., Wang H.C., Chou P.H., Ko W.C. (2015). Clinical implications of species identification in monomicrobial *Aeromonas* bacteremia. PLoS ONE.

[13] Rhee J.Y., Jung D.S., Peck K.R. (2016). Clinical and Therapeutic Implications of *Aeromonas* Bacteremia: 14 Years Nation-Wide Experiences in Korea. Infect. Chemother..

[14] Ko W.C., Lee H.C., Chuang Y.C., Liu C.C., Wu J.J. (2000). Clinical features and therapeutic implications of 104 episodes of monomicrobial *Aeromonas* bacteraemia. J. Infect..

[15] Syue L.S., Chen P.L., Wu C.J., Lee N.Y., Lee C.C., Li C.W., Li M.C., Tang H.J., Hsueh P.R., Ko W.C. (2016). Monomicrobial *Aeromonas* and *Vibrio* bacteremia in cirrhotic adults in southern Taiwan: Similarities and differences. J. Microbiol. Immunol. Infect..

[16] Wu C.J., Chen P.L., Tang H.J., Chen H.M., Tseng F.C., Shih H.I., Hung Y.P., Chung C.H., Ko W.C. (2014). Incidence of *Aeromonas* bacteremia in southern Taiwan: *Vibrio* and *Salmonella* bacteremia as comparators. J. Microbiol. Immunol. Infect..

[17] Ko W.C., Chuang Y.C. (1995). *Aeromonas* Bacteremia: Review of 59 Episodes. Clin. Infect. Dis..

[18] Lau S.M., Peng M.Y., Chang F.Y. (2000). Outcomes of *Aeromonas* bacteremia in patients with different types of underlying disease. J. Microbiol. Immunol. Infect..

[19] Nolla-Salas J., Codina-Calero J., Vallés-Angulo S., Sitges-Serra A., Zapatero-Ferrándiz A., Climent M.C., Gómez J., Masclans J.R. (2017). Clinical significance and outcome of *Aeromonas* spp. Infections among 204 adult patients. Eur. J. Clin. Microbiol. Infect. Dis..

[20] Evans L., Rhodes A., Alhazzani W., Antonelli M., Coopersmith C.M., French C., Machado F.R., Mcintyre L., Ostermann M., Prescott H.C. (2021). Surviving sepsis campaign: International guidelines for management of sepsis and septic shock 2021. Intensive Care Med..

[21] Chang S.H., Hsieh C.H., Weng Y.M., Hsieh M.S., Goh Z.N.L., Chen H.Y., Chang T., Ng C.J., Seak J.C., Seak C.K. (2018). Performance Assessment of the Mortality in Emergency Department Sepsis Score, Modified Early Warning Score, Rapid Emergency Medicine Score, and Rapid Acute Physiology Score in Predicting Survival Outcomes of Adult Renal Abscess Patients in the Emergency Department. Biomed. Res. Int..

[22] Wei X., Ma H., Liu R., Zhao Y. (2019). Comparing the effectiveness of three scoring systems in predicting adult patient outcomes in the emergency department. Medicine.

[23] Hsieh C.C., Yang C.Y., Lee C.H., Chi C.H., Lee C.C. (2020). Validation of MEDS score in predicting short-term mortality of adults with community-onset bacteremia. Am. J. Emerg. Med..

[24] Choi H., Kim J.J., Kim J., Seong H., Lee S.J., Jeong W., Jeong I.Y., Jeong S.J., Ku N.S., Han S.H. (2017). Risk Factors for Mortality in Patients with *Aeromonas* Bacteremia. Open Forum Infect. Dis..

[25] Janda J.M., Brenden R. (1987). Importance of *Aeromonas sobria* in *Aeromonas* Bacteremia. J. Infect. Dis..

[26] Janda J.M., Clark R.B., Brenden R. (1985). Virulence of *Aeromonas* species as assessed through mouse lethality studies. Curr. Microbiol..

[27] Wu C.J., Wu J.J., Yan J.J., Lee H.C., Lee N.Y., Chang C.M., Shih H.I., Wu H.M., Wang L.R., Ko W.C. (2007). Clinical significance and distribution of putative virulence markers of 116 consecutive clinical *Aeromonas* isolates in southern Taiwan. J. Infect..

[28] Chan F.K., Ching J.Y., Ling T.K., Chung S.C., Sung J.J. (2000). *Aeromonas* infection in acute suppurative cholangitis: Review of 30 cases. J. Infect..

[29] Clark N.M., Chenoweth C.E. (2003). *Aeromonas* Infection of the Hepatobiliary System Report of 15 Cases and Review of the Literature. Clin. Infect. Dis..

[30] Ketover B.P., Young L.S., Armstrong D. (1973). Septicemia due to *Aeromonas hydrophila*: Clinical and immunologic aspects. J. Infect. Dis..

[31] Pearson T.A., Mitchell C.A., Hughes W.T. (1972). *Aeromonas hydrophila* septicemia. Am. J. Dis. Child..

[32] Lin R.S., Lee F.Y., Lee S.D., Tsai Y.T., Lin H.C., Lu R.H., Hsu W.C., Huang C.C., Wang S.S., Lo K.J. (1995). Endotoxemia in patients with chronic liver diseases relationship to severity of liver diseases, presence of esophaegeal varices, and hyperdynamic circulation. J. Hepatol..

[33] Wiest R., Lawson M., Geuking M. (2014). Pathological bacterial translocation in liver cirrhosis. J. Hepatol..

[34] Vivas S., Rodriguez M., Palacio M.A., Linares A., Alonso J.L., Rodrigo L. (2001). Presence of bacterial infection in bleeding cirrhotic patients is independently associated with early mortality and failure to control bleeding. Dig. Dis. Sci..

[35] Reverter E., Tandon P., Augustin S., Turon F., Casu S., Bastiampillai R., Keough A., Llop E., González A., Seijo S. (2014). AMELD-based model to determine risk of mortality among patients with acute variceal bleeding. Gastroenterology.

[36] Borzio M., Salerno F., Piantoni L., Cazzaniga M., Angeli P., Bissoli F., Boccia S., Colloredo-Mels G., Corigliano P., Fornaciari G. (2001). Bacterial infection in patients with advanced cirrhosis: A multicenter prospective study. Dig. Liver Dis..

[37] Goulis J., Armonis A., Patch D., Sabin C., Greenslade L., Burroughs A.K. (1998). Bacterial infection is independently associated with failure to control bleeding in cirrhotic patients with gastrointestinal hemorrhage. Hepatology.

[38] Bernard B., Cadranel J.F., Valla D., Escolano S., Jarlier V., Opolon P. (1995). Prognostic significance of bacterial infection in bleeding cirrhotic patients: A prospective study. Gastroenterology.

[39] Zhang G., Zhang K., Zheng X., Cui W., Hong Y., Zhang Z. (2020). Performance of the MEDS score in predicting mortality among emergency department patients with a suspected infection: A meta-analysis. Emerg. Med. J..

[40] Hung S.K., Ng C.J., Kuo C.F., Goh Z.N.L., Huang L.H., Li C.H., Chan Y.L., Weng Y.M., Seak J.C., Seak C.K. (2017). Comparison of the Mortality in Emergency Department Sepsis Score, Modified Early Warning Score, Rapid Emergency Medicine Score and Rapid Acute Physiology Score for predicting the outcomes of adult splenic abscess patients in the emergency department. PLoS ONE.

[41] Smith G.B., Prytherch D.R., Meredith P., Schmidt P.E., Featherstone P.I. (2013). The ability of the National Early Warning Score (NEWS) to discriminate patients at risk of early cardiac arrest, unanticipated intensive care unit admission, and death. Resuscitation.

[42] Almutary A., Althunayyan S., Alenazi K., Alqahtani A., Alotaibi B., Ahmed M., Osman I.S., Kakpuri A., Alanazi A., Arafat M. (2020). National Early Warning Score (NEWS) as Prognostic Triage Tool for Septic Patients. Infect. Drug Resist..

[43] Valcarcel B., De-la-Cruz-Ku G., Malpica L., Enriquez-Vera D. (2021). Clinical features and outcome of *Aeromonas sobria* bacteremia in pediatric and adult patients with hematologic malignancies: A single-center retrospective study in Peru. PLoS ONE.

[44] Shapiro N.I., Howell M.D., Talmor D., Donnino M., Ngo L., Bates D.W. (2003). Mortality in Emergency Department Sepsis (MEDS) score: A prospectively derived and validated clinical prediction rule. Crit. Care Med..

[45] Jiang Y., Jiang F.Q., Kong F., An M.M., Jin B.B., Cao D., Gong P. (2019). Inflammatory anemia-associated parameters are related to 28-day mortality in patients with sepsis admitted to the ICU: A preliminary observational study. Ann. Intensive Care.

[46] Subbe C.P., Kruger M., Rutherford P., Gemmel L. (2001). Validation of a modified Early Warning Score in medical admissions. QJM.

[47] Goodacre S., Turner J., Nicholl J. (2006). Prediction of mortality among emergency medical admissions. Emerg. Med. J..

[48] Olsson T., Terent A., Lind L. (2004). Rapid Emergency Medicine Score: A new prognostic tool for in-hospital mortality in non-surgical emergency department patients. J. Intern. Med..

[49] April M.D., Aguirre J., Tannenbaum L.I., Moore T., Pingree A., Thaxton R.E., Sessions D.J., Lantry J.H. (2017). Sepsis clinical criteria in emergency department patients admitted to an intensive care unit: An external validation study of quick Sequential Organ Failure Assessment. J. Emerg. Med..

