# Supplementary material for: Good Performance of Revised Scoring Systems in Predicting Clinical Outcomes of Aeromonas Bacteremia in the Emergency Department: A Retrospective Observational Study
Source: Diagnostics (Basel). 2024 Jan 5;14(2):124. doi: 10.3390/diagnostics14020124 (PMC10814924; doi:10.3390/diagnostics14020124)
Supplement: Supplementary file 1 [file diagnostics-14-00124-s001.zip › diagnostics-2749354-supplementary.pdf]

Table S1: Scoring systems

Mortality in emergency department sepsis (MEDS) score [43]

Mortality in emergency department sepsis (MEDS) score		Points
1.	Terminal illness with possible death in 1 month	6
2.	Hypoxia or tachypnea	3
3.	Shock from sepsis	3
4.	Platelet count below 150,000	3
5.	Granulocytic bands >5% of white blood cells	3
6.	Patient older than 65 years old	3
7.	Lower respiratory infection	2
8.	Patient is from a nursing home	2
9.	Mental status is altered	2

Modified Early Warning Score [46]

Points	3	2	1	0	1	2	3
Temperature (°C)		35 or less		35.1–38.4		38.5 or higher	
Heart rate (bpm)		39 or less	40–50	51–100	101–110	111–129	130 or higher
Systolic blood pressure (mmHg)	70 or less	71–80	81–100	101–199		200 or higher	
Respiratory rate		8 or less	9	10–18	19–20	21–29	30 or higher
AVPU score				A	V	P	U

National Early Warning Score [41]

Physiological parameters	3	2	1	0	1	2	3
Respiration Rate (breaths per minute)	≤8		9–11	12–20		21–24	≥25
SpO2 (%)	≤91	92–93	94–95	≥96			
Any supplemental oxygen?		Yes		No			
Temperature (°C)	≤35.0		35.1–36.0	36.1–38.0	38.1–39.0	≥39.1	
Systolic BP (mmHg)	≤90	91–100	101–110	111–219			≥220
Heart/pulse rate (beats per minute)	≤40		41–50	51–90	91–110	111–130	≥131
Level of consciousness (AVPU system)				A			V, P or U

Rapid Acute Physiology Score [47]

Variable	0	+1	+2	+3	+4
PR (/min)	70–109		55–69<br>110–139	40–54<br>140–179	≤39<br>≥180
MAP (mmHg)	70–109		50–69<br>110–129	130–159	≤49<br>≥160
RR (/min)	12–24	10–11<br>25–34	6–9	35–49	≤5<br>≥50
GCS	≥14	11–13	8–10	5–7	≤4

Rapid Emergency Medicine Score [48]

REMS scoring system	**Score**						
Variable	0	+1	+2	+3	+4	+5	+6
Age (years)	<45		45–54	55–64		65–74	>74
Mean arterial pressure (mmHg)	70–109		110–129<br>50–69	130–159	>159<br>≤49		
Heart rate (bpm)	70–109		110–139<br>55–69	140–179<br>40–54	>179<br>≤39		
Respiratory rate (breaths/min)	12–24	25–34<br>10–11	6–9	35–49	>49<br>≤5		
O_2 saturation (%)	>89	86–89		75–85	<75		
Glasgow Coma Scale	14 or 15	11–13	8–10	5–7	3 or 4		

quick Sequential Organ Failure Score [49]

	Point
Respiratory Rate > 22	1
Systolic Blood Pressure < 100	1
Glasgow Coma Scale <14	1
